# Longitudinal associations between chronic physical conditions, play behavior, and mental health problems in children

**DOI:** 10.1007/s12519-025-00945-z

**Published:** 2025-07-31

**Authors:** Emmie W. Koevoets, Sanne L. Nijhof, Maartje D. Stutvoet, Remco C. Veltkamp, Liesbeth Duijts, Manon H. J. Hillegers, Pauline W. Jansen

**Affiliations:** 1 Department of Pediatrics, Wilhelmina Children’s Hospital, University Medical Center Utrecht, Utrecht University, PO Box 85090, Utrecht, The Netherlands; 2 Department of Information and Computing Sciences, Faculty of Science, Utrecht University, Utrecht, The Netherlands; 3 Department of Pediatrics, Division of Respiratory Medicine and Allergology, Erasmus MC, University Medical Center Rotterdam, Rotterdam, The Netherlands; 4 Department of Neonatal and Pediatric Intensive Care, Division of Neonatology, Erasmus MC, University Medical Center Rotterdam, Rotterdam, The Netherlands; 5 Department of Child and Adolescent Psychiatry/Psychology, Erasmus MC, University Medical Center Rotterdam, Rotterdam, The Netherlands; 6 The Generation R Study Group, Erasmus MC, University Medical Center Rotterdam, Rotterdam, The Netherlands; 7 Department of Psychology, Education, and Child Studies, Erasmus University Rotterdam, Rotterdam, The Netherlands

**Keywords:** Activity limitations, Chronic condition, Mental health, Pediatrics, Play behavior, Population-based cohort

## Abstract

**Background:**

Children and adolescents with chronic physical conditions have an increased probability of mental health problems, which may be partly attributed to the variations in play behavior**.** This study explores the associations between having a chronic physical condition, play behavior, and having mental health problems.

**Methods:**

Data from the Generation R Study were analyzed, a population-based, prospective cohort in the Netherlands. Chronic physical conditions were typically identified before six years of age. Play behavior was assessed at ages 6 and 10. Mental health problems were measured with the Child Behavior Checklist at 14 years of age. Logistic, ranked-order, and linear regression analyses were used to examine direct associations, and mediation analyses were used to investigate indirect paths. *P* values were false discovery rate adjusted.

**Results:**

Of the 4043 included participants, 691 (17%) had a chronic physical condition. Having a chronic physical condition was associated with more mental health problems and limited activity at the age of six years. Playing sports and engaging in social interactions at age 10 were related to fewer mental health problems at age 14. Limited activity mediated the relationship between having a chronic physical condition and mental health problems (social: mediated_proportion_ = 8.33%, 95% CI = 2.02%; 15.7%, *P*_adj_ < 0.001; physical: mediated_proportion_ = 7.72%, 95% CI = 1.73%; 19.1%, *P*_adj_ = 0.040).

**Conclusions:**

Health-related limitations in social and physical activities mediate the relationship between having a chronic physical condition and mental health problems in children. Participation in social and physical activities early in life may be crucial for the mental well-being of children with a chronic physical condition.

**Graphical abstract:**

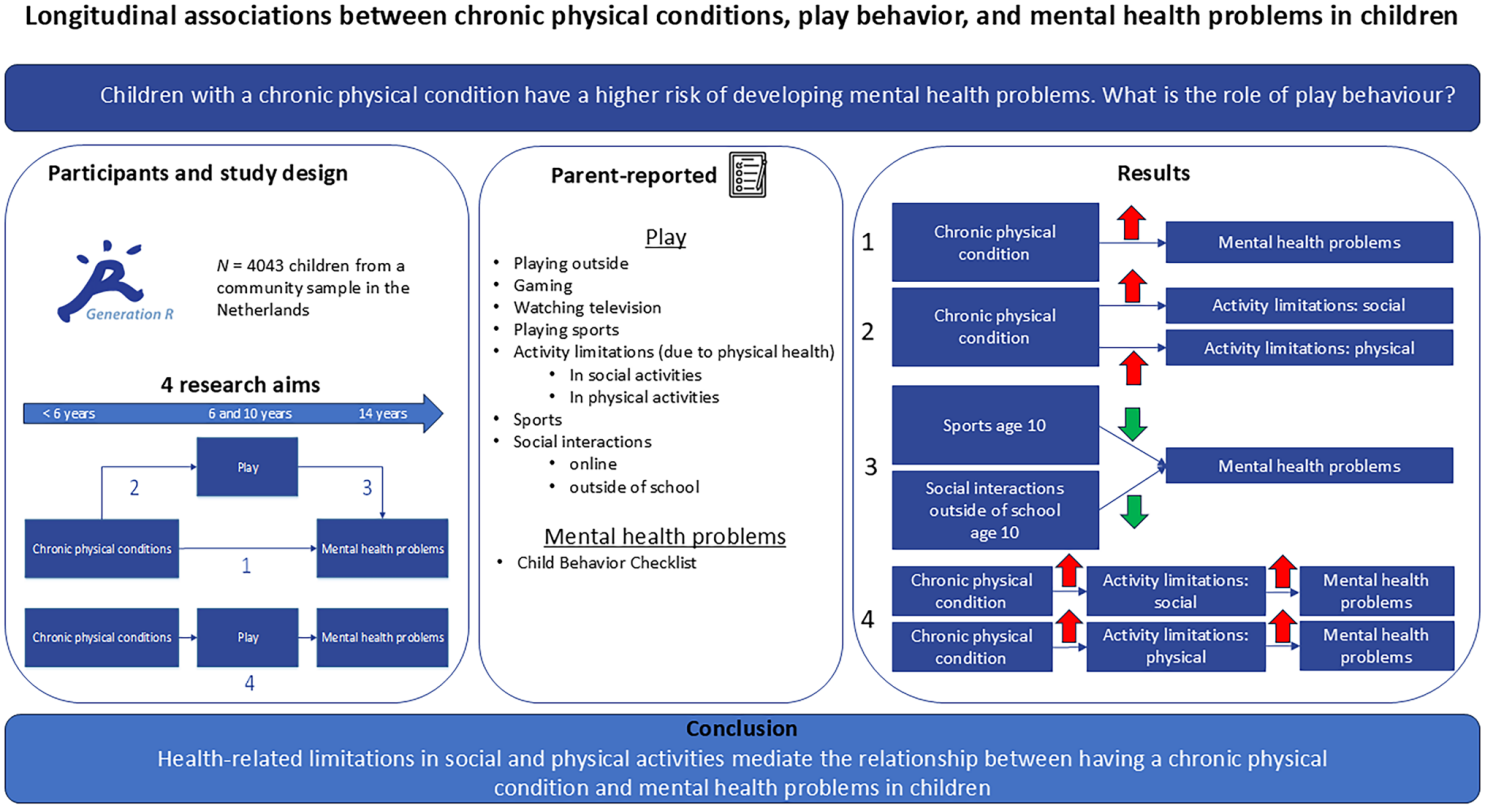

**Supplementary Information:**

The online version contains supplementary material available at 10.1007/s12519-025-00945-z.

## Introduction

In the Netherlands, about 18% of children and adolescents under the age of 25 years live with a chronic physical condition, including illnesses such as asthma, eczema, epilepsy, and congenital heart defects but also handicaps as hearing and visual impairments [1]. Chronic physical conditions have a negative influence on daily life, social functioning and health care costs [2–4], and delay the achievement of (psychosocial) developmental milestones [5]. Moreover, children and adolescents with chronic physical conditions have an increased probability of mental health problems compared with their healthy peers [1, 6, 7]. The mechanisms underlying this association are less well understood, despite the potential to guide the development of interventions aimed at preventing mental health problems in children with chronic physical conditions [8].

Mental health problems in these children may be partly explained by the necessary adjustments in play behavior and/or fewer opportunities to play [9]. Play is essential for healthy brain, behavior, and cognitive development [10–12], and animal research has shown that depriving rats of social play results in social, cognitive, emotional, and sensorimotor deficits [11, 13]. In humans, objectively and consistently studying play behavior is complicated and further exacerbated by obvious ethical constraints preventing the induction of play deprivation [9]. However, healthy play behavior potentially fosters resilience and prepares a child for adult life [9].

In children with a chronic physical condition, play behavior may be restricted by factors such as the disease itself, related stressful events, interpersonal changes (e.g.,social-attachment issues, social interactions with peers, and/or over-anxious parents), and/or social isolation [9]. Consequently, the reduced or altered (social) play behavior in children with a chronic physical condition may contribute in part to the increased risk of experiencing mental health problems [14]. Factors such as spending time outdoors [15], physical activity [16], sports participation [17], and (positive) social interactions all play a role in mental well-being [18]. It remains unclear which and when specific play behavior contributes most to mental well-being and personal development. Insight into the timing and specifics of these contributing factors can help professionals, patients, and parents better understand and support these invaluable processes in childhood and provide clues on how to prevent or alleviate the burden of mental health problems during early adolescence in this already challenged population.

This study aimed to examine prospective associations between having a chronic physical condition, play behavior, and having mental health problems.

## Methods

### Preregistration

This study was preregistered before the analyses were conducted (https://osf.io/ka6n2/). Small adjustments were made during the analysis phase, as described in Supplementary Text 1.

### Design and population

**Fig. 1:**
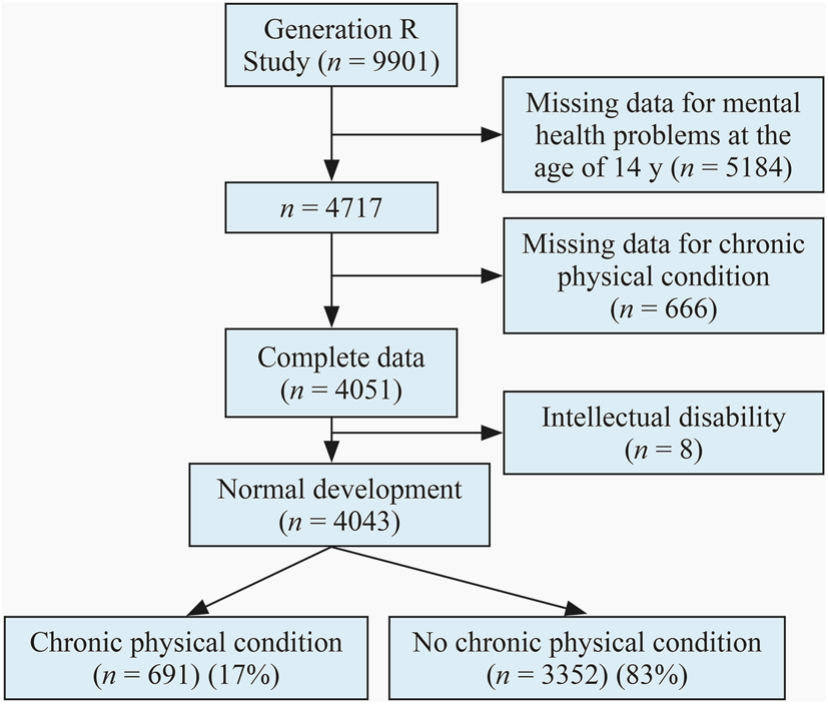
Flow chart of study inclusion

For this study, data from the Generation R Study, a population-based, prospective cohort, were analyzed [19]. The primary aim of this cohort is to investigate the genetic and environmental factors influencing (ab)normal growth, health, and development across childhood into early adulthood. Pregnant women with due dates between April 2002 and January 2006 (and their expected child) were eligible for participation when living in Rotterdam, the Netherlands (participation rate: 61%, *n* = 9901). The study protocol was approved by the medical ethical committee of the Erasmus MC (University Medical Center Rotterdam, Rotterdam, the Netherlands; MEC 217.595/2002/20), and parents (or legal representatives) of all participants provided written informed consent. Measurement waves were conducted during pregnancy, with follow-ups in childhood and adolescence for those with postnatal consent (*n* = 7893). Data on outcomes (mental health problems) at the age of 14 years were missing due to withdrawal, loss to follow-up, or nonresponse on this specific questionnaire (*n* = 5184). Subjects with missing data on chronic physical conditions (*n* = 666) as well as those with an intellectual disability (*n* = 8) were excluded from the analyses (Fig. 1).

### Measures

#### Exposure: chronic physical conditions

In the original assessment of the prevalence of chronic conditions, van Hal et al. included both physical and mental conditions [1]. For this project, we specifically focused on chronic physical conditions. The following conditions were assessed and identified as chronic physical conditions (mostly before the age of six years): eczema, asthma, morbid obesity, vision impairment, hearing impairment, walking impairment, congenital heart disease, epilepsy, celiac disease, irritable bowel syndrome, and persistent constipation (Supplementary Table 1). In addition, an open-ended question at the age of three years was used to supplement the variable chronic physical condition (“Has your child ever been diagnosed by a doctor as having one or more of the following disorders? Chronic sickness, namely..”). Individual subjects were reviewed and discussed by KEW and NSL (pediatrician).

Data for chronic physical conditions were considered missing (and excluded) when data for fewer than four out of the 11 prespecified conditions were available. The available data were used to define chronic physical conditions as a binary variable (yes/no).

#### Mediators: play behavior

Play behavior was parent-reported at the ages of 6 and 10 years. We included measures of the following play behaviors at the age of six years (Supplementary Table 2): playing outside, gaming, watching television (all: average time spent in hours/day), playing sports (no, ≤ 1 hour/week, > 1 hour/week), limited in social activities and physical activities (being restricted in school and social activities due to physical health, being restricted in physical activities due to physical health; yes/no). At the age of 10 years, we defined the following play behaviors: playing outside, gaming, watching television (all: average time spent in hours/day), playing sports (< 1 hour/week, 1–2 hours/week, 2–4 hours/week, > 4 hours/week), social interactions online (chatting and spending time on social network sites; yes/no), and social interactions outside of school (not at all, slightly, quite, extremely).

#### Outcome: mental health problems

Mental health problems of the child were measured at the age of 14 years by the total problem score of the Child Behavior Checklist (CBCL), a component of the Achenbach System of Empirically Based Assessment (ASEBA) [20]. This checklist is a well-validated parent report and consists of 113 items scored on a three-point Likert scale (0 = not true, 1 = somewhat or sometimes true, 2 = very true or often true), where higher scores indicate more emotional and behavioral problems. For boys aged 12–18 years, a score of ≥ 40 is considered borderline problematic, and for girls aged 12–18 years, a score of ≥ 36 is considered borderline problematic. The items can be divided into two broadband scales: internalizing problems and externalizing problems.

### Covariates

Covariates were chosen on the basis of their possible relationships with both play behavior and mental health problems. Age and sex were recorded. Ethnicity was categorized as Dutch, other Western, or non-Western, and maternal educational level was categorized as low (no education/primary school/first phase of secondary school), middle (lower or higher vocational training), or high (university). Maternal psychopathology was self-reported by the mother with the Brief Symptom Inventory (BSI) [21]. The global severity index (GSI) was calculated as the sum score of 53 items, with scores for each item ranging from 1 (not at all) to 5 (continually) on a five-point Likert scale. Ethnicity, education, and maternal psychopathology were assessed during pregnancy.

### Statistical analysis

The demographic characteristics of participants with a chronic physical condition and participants without a chronic physical condition were compared via independent samples t tests for continuous variables and *χ*^*2*^ tests for categorical variables. A nonresponse analysis was conducted to assess potential selection bias. The demographic characteristics of the sample of responders (*n* = 4051) were compared to those of participants with missing data on the exposure and outcome variables (*n* = 5850) via independent samples t tests and *χ*^*2*^ tests. The CBCL total score and continuous play variables were square root transformed variables to meet the normality assumption when included as outcome measures in the analyses. Beta values represent unstandardized effect estimates. Multiple imputation procedures (MICE package, R) were used to address missing values in play behavior and covariates [22]. The variable with the most missing values was social interactions outside of school (23%); therefore, 25 datasets were imputed [23].

The first aim was to investigate the direct associations between having a chronic physical condition, engaging in play behavior, and having mental health problems. We used logistic, ranked-order, and linear regression analyses for binary, ordinal, and continuous outcome variables, respectively. All covariates were included in all the models. In addition, the CBCL total problem score at the age of six years was included as a covariate in the models investigating the direct relationship between play behavior and mental health problems at the age of 14 years to correct for a possible reverse causal effect of mental health problems on play behavior.

Second, we investigated whether certain play behaviors mediated the relationship between having a chronic physical condition and mental health problems. Mediation analyses were performed only if there was a significant relationship between chronic physical condition and mental health problems as well as between having a chronic physical condition and certain play behavior (*P*_uncorrected_ < 0.05). The proportion of mediation was calculated via regression-based mediation analyses, which were corrected for all covariates and were conducted via the R package CMAverse [24]. We used the default setting for bootstrapping to calculate the 95% confidence intervals (CIs).

Exploratory and sensitivity analyses were conducted for the direct associations. First, if there was a significant relationship between having a chronic physical condition and mental health problems, we conducted a post hoc analysis to determine whether having a chronic physical condition was associated primarily with internalizing problems or externalizing problems. Second, we determined whether participants had a borderline/clinical total score on the CBCL (which corresponded with the ≥ 83rd percentile or a T score ≥ 60). We then repeated the analyses via this binary CBCL outcome measure and applied quasi-Poisson regressions to generate risk ratios (RRs) for improved interpretability. Third, all analyses were repeated with the CBCL measured at the age of 10 years as the outcome to investigate the robustness of our results. Fourth, we repeated the analyses with a broader definition for having a chronic physical condition, complementing the chronic physical condition definition by including the following diagnoses: (food) allergies, lactose intolerance, and a few individual subjects on the basis of the open-ended question about chronic illness asked at the age of three years. Finally, we repeated the analysis investigating the association between having a chronic physical condition and mental health problems after excluding 14 items from the CBCL total score that may directly relate to the chronic physical condition and not necessarily to mental health problems. This included items from the somatic complaints scale and individual items involving overeating, overweight, and bowel movements outside the toilet.

All *P* values were corrected for the false discovery rate (FDR) via the Benjamini‒Hochberg procedure and considered significant when they were < 0.05. All analyses were conducted via R statistical software packages [25].

### Declaration of generative artificial intelligence and artificial intelligence-assisted technologies in the writing process

During the preparation of this work, the authors used ChatGPT to improve language and readability. After using this tool/service, the authors reviewed and edited the content as needed and take full responsibility for the content of the publication.

## Results

### Participant characteristics

We included 4043 participants in the analyses, of whom 691 (17.1%) had a chronic physical condition and 3352 (82.9%) did not (Table 1). Participants with a chronic physical condition had, on average, a lower birth weight (mean difference = 77.3 g, *P* = 0.001), and their mothers experienced more psychopathology during pregnancy (mean difference score = 0.051, *P* < 0.001). There were no additional demographic differences between participants with or without a chronic physical condition.

**Table 1:** Descriptive statistics of participants

Characteristics	Total	Chronic physical condition	No chronic physical condition	*P*
*n*	4043 (100)	691 (17.1)	3352 (82.9)	
Sex, *n* (%)				1.000
Boy	1999 (49.4)	342 (49.5)	1657 (49.4)	
Girl	2044 (50.6)	349 (50.5)	1695 (50.6)	
Ethnicity, *n* (%)				0.073
Dutch	2792 (69.3)	460 (66.8)	2332 (69.9)	
Western	349 (8.7)	55 (8.0)	294 (8.8)	
Non-Western	886 (22.0)	174 (25.3)	712 (21.3)	
Age at 14 y measuring point, mean (SD)	13.5 (0.37)	13.5 (0.40)	13.5 (0.36)	0.469
Mental health problems (CBCL), *n* (%)				0.078
Yes	449 (11.1)	90 (13.0)	359 (10.7)	
No	3594 (88.9)	601 (87.0)	2993 (89.3)	
Mother age at birth, mean (SD)	31.8 (4.48)	31.7 (4.52)	31.8 (4.47)	0.560
Birth weight (g), mean (SD)	3438 (580)	3374 (630)	3451 (568)	0.001*
Maternal education level, *n* (%)				0.757
Low	487 (12.7)	88 (13.5)	399 (12.5)	
Middle	2061 (53.5)	345 (53.1)	1716 (53.6)	
High	1301 (33.8)	217 (33.4)	1084 (33.9)	
Maternal psychopathology (GSI), mean (SD)	0.22 (0.28)	0.26 (0.35)	0.21 (0.26)	< 0.001^†^
Chronic physical conditions^a^, *n* (%)				-
Asthma	178 (4.4)	178 (25.8)		
Congenital heart disease	19 (0.47)	19 (2.75)		
Epilepsy	2 (0.05)	2 (0.29)		
Morbid obesity	13 (0.32)	13 (1.88)		
Irritable bowel syndrome	35 (0.87)	35 (5.07)		
Celiac disease	23 (0.57)	23 (3.33)		
Persistent constipation	62 (1.53)	62 (8.97)		
Sight impaired	12 (0.30)	12 (1.74)		
Hearing impaired	3 (0.07)	3 (0.43)		
Walking impaired	2 (0.05)	2 (0.29)		
Other conditions	12 (0.28)	12 (1.74)		

Values are based on unimputed data. *P* values indicate group differences. * *P* value < 0.01. † *P* value < 0.001

^a^Chronic physical conditions are not mutually exclusive, except for the category “other conditions”. The category “other conditions” represented unique subjects without any additional chronic physical condition and included epidermolysis bullosa, familial hypercholesterolemia, Hirschsprung’s disease, juvenile idiopathic arthritis, Von Recklinghausen’s disease, retinoblastoma, thyroid disease, Von Willebrand disease, and congenital diaphragmatic hernia

- not available, *CBCL* Child Behavior Checklist, *GSI* Global Severity Index of the Brief Symptom Inventory, *SD* standard deviation

The results of the nonresponse analysis indicated that participants were less inclined to respond when they were male (*χ*^*2*^ = 3.86, *P* = 0.049) or non-Dutch (*χ*^*2*^ = 789, *P* < 0.001), had a lower birth weight (mean difference = − 88 g, *P* < 0.001), or when their mothers completed a lower level of education (*χ*^*2*^ = 835, *P* < 0.001), were younger (mean difference = 3.22 years, *P* < 0.001), or reported higher levels of psychopathology (mean difference score = 0.16, *P* < 0.001).

### Direct relations: chronic physical conditions, play behavior, and mental health problems

Several small direct associations between having a chronic physical condition, play behavior, and mental health problems remained significant after adjusting for multiple testing [range partial *R*^*2*^: 0.00313 (chronic physical condition)–0.00999 (social interactions outside of school)] (Table 2). Having a chronic physical condition was associated with a higher CBCL total problem score, indicating mental health problems [chronic physical condition: mean CBCL = 20.0 (SD = 16.4); no chronic physical condition: mean = 17.4 (SD = 15.8); *β* = 0.27, 95% CI = 0.13; 0.42, *P*_adj_ = 0.002]. Among participants with chronic physical conditions, 13.0% had at least a borderline problematic score on the CBCL. The percentage of having at least a borderline problematic score on the CBCL in participants without a chronic physical condition was 10.7% (*P* = 0.078).

**Table 2:** Associations of having a chronic physical condition and play behavior with mental health problems at the age of 14 years

Variables	Estimate for CBCL total problem score (95% CI)	*P*_uncorrected_	*P*_adjusted_	Effect size partial *R*^*2*^
Chronic physical condition				
No	Reference			
Yes	0.27 (0.13; 0.42)	< 0.001^‡^	0.002^†^	0.00313
Playing outside (h/d)				
Age 6	0.00 (− 0.05; 0.05)	0.869	0.927	0.00004
Age 10	− 0.04 (− 0.12; 0.04)	0.328	0.584	0.00022
Gaming (h/d)				
Age 6	0.01 (− 0.10; 0.13)	0.840	0.927	0.00003
Age 10	0.06 (− 0.02; 0.14)	0.116	0.265	0.00063
Watching television (h/d)				
Age 6	− 0.03 (− 0.09; 0.03)	0.387	0.589	0.00018
Age 10	0.01 (− 0.04; 0.06)	0.622	0.780	0.00008
Playing sports				
Age 6				
No	Reference			
≤ 1 h/wk	− 0.04 (− 0.16; 0.08)	0.532	0.740	
> 1 h/wk	0.05 (− 0.09; 0.18)	0.516	0.740	0.00024
Age 10				
< 1 h/wk	Reference			
1–2 h/wk	− 0.24 (− 0.52; 0.03)	0.080	0.255	
2–4 h/wk	− 0.34 (− 0.61; − 0.07)	0.013^*^	0.046^*^	
> 4 h/wk	− 0.47 (− 0.75; − 0.20)	< 0.001^‡^	0.004^†^	0.00344
Activity limitations age 6				
Social				
No	Reference			
Yes	0.18 (− 0.04; 0.40)	0.113	0.265	0.00055
Physical				
No	Reference			
Yes	0.12 (− 0.08; 0.31)	0.240	0.513	0.00032
Social interactions age 10				
Online				
No	Reference			
Yes	0.10 (− 0.02; 0.21)	0.096	0.265	0.00062
Outside of school				
Not at all	Reference			
Slightly	− 0.25 (− 0.56; 0.06)	0.108	0.265	
Quite	− 0.54 (− 0.84; − 0.24)	< 0.001^‡^	0.003^†^	
Extremely	− 0.71 (− 1.02; − 0.40)	< 0.001^‡^	< 0.001^‡^	0.00999

CBCL outcome was square root transformed. All models were linear regression analyses adjusted for age at the measuring point of the CBCL, sex, ethnicity, maternal educational level, and maternal psychopathology. Moreover, models involving play behaviors as predictors were also adjusted for CBCL score measured at the age of 6 years

The effect size partial *R*^*2*^ represents the explained variance of the CBCL score by the specified predictor (having a chronic physical condition or a play behavior) in the presence of the covariates. The effect size measure partial *R*^*2*^ can be interpreted as small (0.02), medium (0.13), or large (0.26) [26]

^*^P < 0.05. † *P* < 0.01. ‡ *P* < 0.001

*CBCL* Child Behavior Checklist, *CI* confidence interval

Having a chronic physical condition was associated with being more limited in social activities [odds ratio (OR): 2.00, 95% CI = 1.47; 2.71, *P*_adj_ < 0.001] and physical activities (OR: 2.12, 95% CI = 1.61; 2.78, *P*_adj_ < 0.001) at the age of six years (Table 3). We found no direct relationships between having a chronic physical condition and other play behavior [playing outside (age 6/age 10), gaming (age 6/age 10), watching television (age 6/age 10), playing sports (age 6/age 10), or social interactions (age 10: online/outside of school)].

**Table 3:** Association between having a chronic physical condition and play behavior

Variables	Estimate for having a chronic physical condition on each play behavior (95% CI)	*P*_uncorrected_	*P*_adjusted_	Effect size partial *R*^*2*^ or odds ratio (95% CI)
Playing outside (h/d)^a^				
Age 6	− 0.00 (− 0.04; 0.04)	0.974	0.974	0.00003
Age 10	− 0.01 (− 0.05; 0.02)	0.373	0.589	0.00028
Gaming (h/d)^a^				
Age 6	− 0.01 (− 0.04; 0.02)	0.634	0.780	0.00008
Age 10	− 0.02 (− 0.05; 0.02)	0.296	0.584	0.00036
Watching television (h/d)^a^				
Age 6	− 0.00 (− 0.03; 0.03)	0.805	0.920	0.00003
Age 10	− 0.01 (− 0.04; 0.03)	0.762	0.903	0.00009
Playing sports^b^				
Age 6	− 0.04 (− 0.21; 0.12)	0.616	0.780	0.96 (0.81; 1.13)
Age 10	− 0.08 (− 0.25; 0.08)	0.319	0.584	0.92 (0.78; 1.09)
Activity limitations age 6^c^				
Social	0.69 (0.39; 1.00)	< 0.001^*^	< 0.001^*^	2.00 (1.47; 2.71)
Physical	0.75 (0.48; 1.02)	< 0.001^*^	< 0.001^*^	2.12 (1.61; 2.78)
Social interactions age 10				
Online^c^	0.10 (− 0.11; 0.30)	0.355	0.589	1.10 (0.90; 1.35)
Outside of school^b^	0.01 (− 0.16; 0.17)	0.930	0.960	1.01 (0.85; 1.19)

All models were adjusted for age at the measuring point of the outcome variable (age 6 or 10 years), sex, ethnicity, maternal educational level, and maternal psychopathology

Note: for interpretability, risk ratios (RRs) based on Poisson regression with robust standard errors are provided for binary outcomes: activity limitations age 6–social: RR = 1.85 (95% CI: 1.38; 2.47); activity limitations age 6–physical: RR = 1.89 (95% CI: 1.47; 2.42); social interactions age 10–online: RR = 1.06 (95% CI: 0.91; 1.24)

^a^Linear regression analyses with partial *R*^*2*^ as an effect size measure. The effect size partial *R*^*2*^ represents the explained variance of the specified play behavior by having a chronic physical condition in the presence of the covariates. The effect size measure partial *R*^*2*^ can be interpreted as small (0.02), medium (0.13), or large (0.26) [26]

^b^For ranked-order regression analyses, we used a proportional odds ratio (OR) as an effect size measure

^c^For logistic regression analyses, we used an OR as an effect size measure

*P < 0.001

*CI* confidence interval

A positive impact of play behavior on mental health problems was found for playing sports at the age of 10 years (compared with < 1 hour/week, 2–4 hours/week: *β* = − 0.34, 95% CI = − 0.61; − 0.07, *P*_adj_ = 0.046; > 4 hours/week: *β* = − 0.47, 95% CI = − 0.75; − 0.20, *P*_adj_ = 0.004) as well as social interactions outside of school (compared with ‘not at all’, quite: *β* = − 0.54, 95% CI = − 0.84; − 0.24, *P*_adj_ = 0.003; extremely: *β* = − 0.71, 95% CI = − 1.02; − 0.40, *P*_adj_ < 0.001) (Table 2). We found no significant relationships between other play behavior [playing outside (age 6/age 10), gaming (age 6/age 10), watching television (age 6/age 10), playing sports (age 6/age 10: < 2 hours/week), activity limitations (age 6: social/physical), or social interactions (age 10: online)] and mental health problems.

### Mediation analyses

**Fig. 2:**
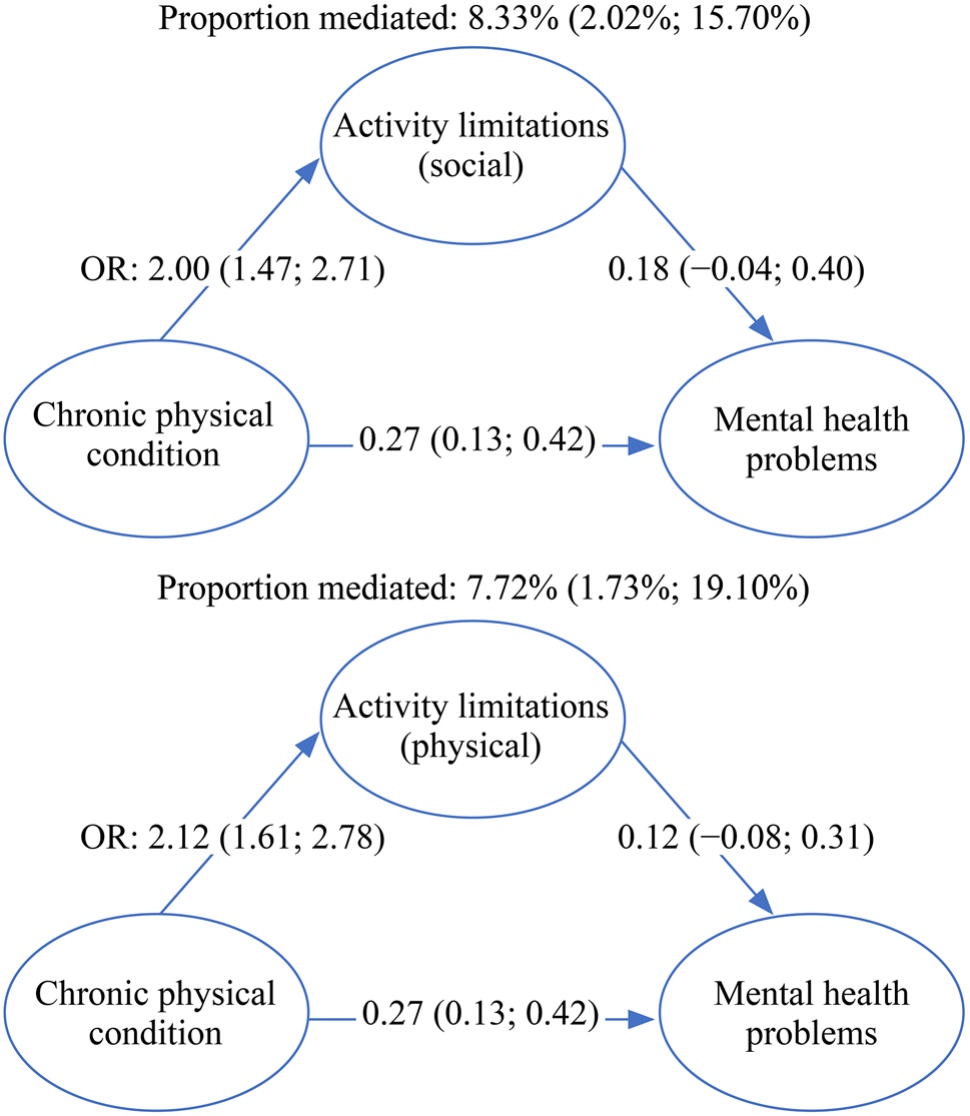
Results of the mediation analyses. The values represent the estimates and 95% confidence intervals (CI) unless indicated otherwise. Note: mediation analyses were performed in two separate models. Both models were adjusted for age at the measuring point of the outcome variable, sex, ethnicity, maternal educational level, and maternal psychopathology. *OR* odds ratio

Analyses were conducted for the mediating factors of limited social and physical activity, both of which were measured at the age of six years (Fig. 2). Both limited social activity (mediated_proportion_ = 8.33%, 95% CI = 2.02; 15.7, *P*_adj_ < 0.001) and limited physical activity (mediated_proportion_ = 7.72%, 95% CI = 1.73; 19.1, *P*_adj_ = 0.040) mediated the relationship between having a chronic physical condition and mental health problems.

### Exploratory and sensitivity analyses

First, post hoc analyses revealed that the relationships between having a chronic physical condition and mental health problems were significant for both internalizing problems (*β* = 0.15, 95% CI = 0.05; 0.25, *P* = 0.002) and externalizing problems (*β* = 0.13, 95% CI = 0.03; 0.23, *P* = 0.015).

Second, we repeated the analyses using a binary CBCL outcome indicating borderline/clinical problems. Although the results were in the same direction as those of the original analyses, the association between chronic physical condition and the risk of having borderline problems was not statistically significant (RR: 1.12, 95% CI = 0.90; 1.40, *P* = 0.322) (Supplementary Table 3).

Third, we repeated the analyses with the CBCL measured at the age of 10 years as the outcome to test the robustness of our results. Similar results were found (Supplementary Table 4), with an additional significant positive association between television watching at the age of 10 years and mental health problems (*β* = 0.07, 95% CI = 0.02; 0.12, *P*_adj_ = 0.014). The association between playing sports at 10 years and having fewer mental health problems measured at 10 years showed a trend toward significance (> 4 hours/week: *β* = − 0.28, 95% CI = − 0.53; − 0.03, *P*_adj_ = 0.099).

Finally, repeating the analyses using a broader definition of chronic physical conditions [*n* = 794 (19.6%), *β* = 0.28, 95% CI = 0.14; 0.42, *P* < 0.001] and repeating the analyses using the CBCL score without somatic items (*β* = 0.25, 95% CI = 0.11; 0.39, *P* < 0.001) resulted in similar results as the original analysis between having a chronic condition and mental health problems.

## Discussion

In this large population-based study, we showed that having a chronic physical condition during childhood was related to more mental health problems in early adolescence. In addition, the presence of a chronic physical condition was associated with limitations in social and physical activities, likely attributed to the child’s physical health. We observed that being limited in social and physical activities at the age of six years mediated the association between having a chronic physical condition and mental health problems later in life. The findings were independent of mental health problems at the age of six years.

The relationship between having a chronic physical condition and mental health, as seen in the current study, has been reported previously [1, 6, 7]. Several factors may contribute to the risk of developing mental health problems in children with a chronic physical condition, including age of onset, sex, type of chronic condition, parenting style, and ability to cope with the consequences of having a chronic physical condition [7, 26, 27]. In addition to the factors previously mentioned, Adams, Chien, and Wisk (2019) and the current study demonstrated that activity limitations mediate the relationship between having a chronic physical condition and developing mental health conditions [28]. The current prospective study, which was conducted in the Netherlands, further explored this subject and expanded on these findings with respect to the role of other play behaviors. We showed that being restricted in certain activities due to challenges in physical health was part of the reason why having a chronic physical condition impacted mental health into early adolescence. This measure of activity limitation may reflect the severity of the impact of chronic physical conditions on a child’s mental well-being later in life. Future interventions should focus on reducing activity limitations for vulnerable children with chronic conditions to reduce the probability of developing mental health problems during adolescence.

In addition to the impact of chronic physical conditions, we identified relationships between certain play behaviors and mental health problems. Engaging more in sports at the age of 10 years and social interactions outside of school at the age of 10 years were related to a lower prevalence of mental health problems at the age of 14 years. It is not without reason that play is one of the fundamental rights of children [International Play Association (IPA World)]. In mammals, positive effects of play behavior and negative effects after being restricted in play behavior have been described [10, 11]. In the present study, we observed that, at the age of 10 years, playing sports and interacting more with peers outside of school were associated with fewer mental health problems in early adolescence. Importantly, we observed these associations even while using rather crude (parent-reported questionnaires) measures of play behavior over a follow-up period of four years and while adjusting for preexisting mental health problems, suggesting that our findings are quite robust. Earlier studies reported similar results. Eime et al. (2013) also reported the psychological and social benefits of participating in sports [17]. In addition, the role of social support in psychologic well-being has been described before, given that social interactions are positive [18]. Our results, along with those of previous research, indicate that play behavior (and being restricted in it) during childhood is important for mental health years later.

Some limitations of this study deserve consideration. First, the open-ended question asking parents whether their child had a chronic condition, and if so, specifying the condition, seemed unreliable when considered by itself. Consequently, we opted to use this question as a supplementary inquiry, alongside more specific questions, to better define chronic physical conditions. As a result, this approach added only 12 additional participants to the group of "chronic physical conditions". In our study sample, 17% of the children were identified as having a chronic physical condition, which is comparable to the 18% reported by van Hal et al. (2019) upon which our definitions were based [1]. We believe that our approach minimized missing data on chronic physical conditions, rendering our sample of children with chronic physical conditions representative of the entire population. Moreover, several sensitivity analyses, including a sensitivity analysis using a broader definition of chronic physical conditions, did not yield materially different results, confirming the robustness of our findings. However, the outcome of our nonresponse analysis did indicate a relatively healthy sample, with selective drop-out. In addition, we do not have information regarding the presence of a chronic physical condition in our sample after the age of six years. Furthermore, the effects observed in this study were small, and when a binary outcome variable was used in a sensitivity analysis (to improve interpretability), we found no significant association between having a chronic physical condition and mental health problems. Although even small effect sizes can reflect substantial individual differences, it remains crucial to exercise caution when generalizing the results. Second, play behavior is challenging to define. While we had parent-reported information on different types of play behavior (including playing outside, gaming, and playing sports), we lacked information on the children’s preferred playstyle, such as imaginary play or body play [9]. There might be a relationship between a child’s play style and mental well-being. However, unfortunately, we cannot distinguish, for example, whether a child who plays outside prefers body play or imaginary play. Different play styles might be differently affected by chronic conditions. In future research, incorporating child-reported measures when feasible is recommended. In addition, exploring various play styles is essential for distinguishing the importance of different aspects (such as physical and social components) of play behavior in relation to chronic conditions and mental well-being. While we had to address several limitations in the current study, the sample size and longitudinal character of this cohort design make it a unique opportunity to study the concept of play, which can be considered a major strength of the study.

In conclusion, in a prospective population-based cohort study, although the effect sizes were small with a continuous outcome, we confirmed the association between having a chronic physical condition in childhood and mental health problems in early adolescence, and this relationship was mediated by being restricted in social and physical activities at an earlier age. In addition, playing sports and engaging in social interactions outside of school emerged as predictors of better mental health at a later age. Health behavior in childhood, including being restricted from participating in daily activities, appears to be important for mental health in later years. Future research should prioritize investigating whether the identified associations are consistent across all chronic conditions and differ between various play styles. Future interventions should focus on enabling vulnerable children to participate in society as much as possible.

## Supplementary Information

Below is the link to the electronic supplementary material.

Supplementary file1 (DOCX 57 KB)

## Data Availability

The datasets generated during and/or analyzed during the current study are available from the corresponding author upon reasonable request.
