# Supplementary material for: Longitudinal associations between chronic physical conditions, play behavior, and mental health problems in children
Source: World J Pediatr. 2025 Jul 31;21(8):800–10. doi: 10.1007/s12519-025-00945-z (PMC12380855; doi:10.1007/s12519-025-00945-z)
Supplement: Supplementary file 1 — Supplementary file1 (DOCX 57 KB) [file 12519_2025_945_MOESM1_ESM.docx]

- **Supplementary Text 1** Small deviations from original preregistration
- **Supplementary Table 1** Definitions of included chronic physical conditions
- **Supplementary Table 2** Definitions of included play behaviors
- **Supplementary Table 3** Associations of having a chronic physical condition and play behaviors with a binary measure of mental health problems at age 14 years, a sensitivity analysis
- **Supplementary Table 4** Associations of having a chronic physical condition and play behaviors with mental health problems at age 10 years, a sensitivity analysis
- **References**

**Supplementary Text 1** Small deviations from original preregistration

- After receiving some additional recommendations regarding the mediation analysis from the research group, we decided to execute mediation analyses for significant relations found in aims 1 and 2, instead of all significant relations in aims 1, 2, and 3. Additionally, we used the R-package CMAverse instead of the R-package ‘mediation’.
- For this longitudinal project, we opted to exclusively utilize the Child Behavior Checklist (CBCL) administered at the age of 14 years as the primary outcome measure, rather than using both the CBCL at the age of 14 and 10 years as primary outcome measures. This decision is more sensible since the primary objective of including the CBCL at the age of 10 years in our study was to test the robustness of our results. Additionally, focusing primarily on the CBCL at the age of 14 years would enhance the manuscript’s readability. Therefore, we conducted sensitivity analyses using the CBCL at the age of 10 years. Consequently, p-values that were corrected for false discovery rate did not include the CBCL at the age of 10 years.

**Supplementary Table 1** Definitions of included chronic physical conditions

| **Chronic condition** | **N (%)** | **Question/measurement** | **Definition/answer** | **Age at measurement** |
| --- | --- | --- | --- | --- |
| Asthma | 178 (4.4) | Questionnaire:  Was your child ever diagnosed with asthma by a doctor? | Yes (vs. no) | 6 years |
| Eczema | 423 (10.5) | Questionnaire:   1. Has your child ever had eczema? 2. Was your child seen by a doctor in the past year because of these complaints? | 1. Yes (vs. no) 2. Yes (vs. no) | 6 years |
| Congenital Heart Disease | 19 (0.47) | Questionnaire:  Has your child ever been diagnosed by a doctor with a   - congenital heart condition? | Yes (vs. no) | 2/3 years |
| Epilepsy | 2 (0.05) | Patient information system | Epilepsy diagnosis [1] | 1, 2, 6 years |
| Morbid obesity | 13 (0.32) | Height and weight repeatedly measured at our research centre and child health centres | Of the five repeated BMI measurements (between 2 and 6 years):   - 1 measurement morbid obesity: > SDS of 3.5 - All other measurements at least overweight: > SDS of 1 | 2-6 years |
| Irritable Bowel Syndrome | 35 (0.87) | Questionnaire:  Has your child been diagnosed by a doctor with …   - Irritable Bowel Syndrome or spastic colon | Yes (vs. no) | 10 years* |
| Celiac disease | 23 (0.57) | Questionnaire:  Has your child been diagnosed by a doctor with …   - Coeliac (gluten intolerance) | Yes (vs. no) | 10 years* |
| Persistent constipation | 62 (1.53) | Questionnaire, repeated:  Has your child had constipation, diagnosed by a doctor, for a period of at least 2 weeks in the last year? | Yes for at least 2 out of 4 repeated measurements | 2-6 years |
| Sight impaired | 12 (0.30) | Questionnaire on sight  My child… | Cannot see well, neither close by nor in the distance, even when wearing glasses; Cannot see at all (vs. Can see normally without glasses; Can see normally with glasses; Can see objects that are close quite well, but has limitations seeing things in the distance, even when wearing glasses) | 3 years |
| Hearing impaired | 3 (0.07) | Questionnaire on hearing  My child… | Cannot hear what is said even with a hearing aid; Cannot hear at all (vs. Hears what is said without a hearing aid in normal situations where there is background noise and other distractions; Hears what is said in quiet situations where there are no distractions (with or without a hearing aid); Can hear what is said to a limited extent even with a hearing aid) | 3 years |
| Walking impaired | 2 (0.05) | Questionnaire on walking and moving around  My child… | Cannot move around independently, needs someone’s help and possibly an aid as well, a buggy for example, to move around (vs. Walks, bends over, lifts, jumps and runs just as well as other children of the same age; Has some limitations when walking, bending over, lifting, jumping or running but does not need aids or someone’s assistance to move around independently; Walks or move around without anyone’s assistance but does need aids) | 3 years |
| Other conditions | 12 (0.28) | Questionnaire:  Has your child ever been diagnosed by a doctor with …   - Chronic sickness, namely: | Responses to open-ended question (for cases not yet classified as having a chronic condition):   - Epidermolysis bullosa - Familial hypercholesterolemia - Hirschsprung’s disease - Juvenile idiopathic arthritis - Von Recklinghausen’s disease - Retinoblastoma - Thyroid disease - Von Willebrand disease - Congenital diaphragmatic hernia | 2/3 years |
| Total | 690 (17.1) |  |  |  |

* We aimed at identifying chronic conditions before the age of 6. However, for some conditions, we argued that including a measurement at a later age was preferred over not including the condition at all

**Supplementary Table 2** Definitions of included play behaviors

| **Play behavior** | **Age at measurement** | **Questions and answers** | **Definition** |
| --- | --- | --- | --- |
| Playing outside | 6 | “On average, how many days during the week does your child play outside?”   - - Never on weekdays (coded as 0)   - 1 day per week (coded as 1)   - 2 days per week (coded as 2)   - 3 days per week (coded as 3)   - 4 days per week (coded as 4)   - Every weekday (coded as 5)   “On average, how many weekend days in a week does your child play outside?”   - - Never in the weekend (coded as 0)   - 1 day in the weekend (coded as 1)   - 2 days in the weekend (coded as 2)   “On the days that your child plays outside, how long, on average, does your child then play outside? Differentiate between weekdays and weekends and answer according to the present season.“  *Separate questions for weekdays and weekends, and separately for mornings, afternoons, and evenings after dinner:*   - - Never (coded as 0)   - Less than 30 minutes (coded as 0.25)   - 30-60 minutes (coded as 0.75)   - 1-2 hours (coded as 1.5)   - 2-3 hours (coded as 2.5)   - 3-4 hours (coded as 3.5) | Average time spent playing outside per day =  (days during week/7 * (time spent in morning + afternoon + evening))  + (days in weekend/7 * (time spent in morning + afternoon + evening)) |
| Playing outside | 10 | “On average how many days during the week does your child play outside?”   - - Never (coded as 0)   - 1 or 2 days per week (coded as 1.5)   - 3 or 4 days per week (coded as 3.5)   - 5 or more days per week (coded as 6)   “Approximately how long does your child approximately play outside per day? Only consider the days that your child plays outside.”   - - Less than 30 minutes per day (coded as 0.25)   - 30 minutes to 1 hour per day (coded as 0.75)   - 1 to 2 hours per day (coded as 1.5)   - 2 to 3 hours per day (coded as 2.5)   - 3 to 4 hours per day (coded as 3.5)   - More than 4 hours per day (coded as 4.5) | Average time spent playing outside per day =  days total week/7 * time per day |
| Gaming | 6 | “On average, how many days during the week does your child use the (game)computer?”   - - Never on weekdays (coded as 0)   - 1 day per week (coded as 1)   - 2 days per week (coded as 2)   - 3 days per week (coded as 3)   - 4 days per week (coded as 4)   - Every weekday (coded as 5)   “On average, how many weekend days in a week does your child use the (game)computer?”   - - Never in the weekend (coded as 0)   - 1 day in the weekend (coded as 1)   - 2 days in the weekend (coded as 2)   “On the days that your child uses the (game)computer, how long does he/she use it, on average? Please differentiate here between weekdays and weekends.”  *Separate questions for weekdays and weekends, and separately for mornings, afternoons, and evenings after dinner*   - - Never (coded as 0)   - Less than 30 minutes (coded as 0.25)   - 30-60 minutes (coded as 0.75)   - 1-2 hours (coded as 1.5)   - 2-3 hours (coded as 2.5)   - 3-4 hours (coded as 3.5) | Average time spent gaming per day =  (days during week/7 * (time spent in morning + afternoon + evening))  + (days in weekend/7 * (time spent in morning + afternoon + evening)) |
| Gaming | 10 | “On average, how many days during the week does your child use the (game)computer?”   - - Never on weekdays (coded as 0)   - 1 day per week (coded as 1)   - 2 days per week (coded as 2)   - 3 days per week (coded as 3)   - 4 days per week (coded as 4)   - Every weekday (coded as 5)   “On average, how many weekend days in a week does your child use the (game)computer?”   - - Never in the weekend (coded as 0)   - 1 day in the weekend (coded as 1)   - 2 days in the weekend (coded as 2)   “On the days that your child uses the (game)computer, how long does he/she use it, on average? Please differentiate here between weekdays and weekends.  *Separate questions for weekdays and weekends, and separately for mornings, afternoons, and evenings after dinner*   - - Never (coded as 0)   - Less than 30 minutes (coded as 0.25)   - 30-60 minutes (coded as 0.75)   - 1-2 hours (coded as 1.5)   - 2-3 hours (coded as 2.5)   - 3-4 hours (coded as 3.5) | Average time spent gaming per day =  (days during week/7 * (time spent in morning + afternoon + evening))  + (days in weekend/7 * (time spent in morning + afternoon + evening)) |
| Watching Television | 6 | “On average, how many days during the week does your child watch television/video/DVD?”   - - Never on weekdays (coded as 0)   - 1 day per week (coded as 1)   - 2 days per week (coded as 2)   - 3 days per week (coded as 3)   - 4 days per week (coded as 4)   - Every weekday (coded as 5)   “On average, how many weekend days in a week does your child watch television/video/DVD?”   - - Never in the weekend (coded as 0)   - 1 day in the weekend (coded as 1)   - 2 days in the weekend (coded as 2)   “On the days that your child watches television/video/DVD, how long does he watch, on average? Differentiate here between weekdays and weekends. Also, mornings/afternoons/evenings separately. “  *Separate questions for weekdays and weekends, and separately for mornings, afternoons, and evenings after dinner*   - - Never (coded as 0)   - Less than 30 minutes (coded as 0.25)   - 30-60 minutes (coded as 0.75)   - 1-2 hours (coded as 1.5)   - 2-3 hours (coded as 2.5)   - 3-4 hours (coded as 3.5) | Average time spent watching television per day =  (days during week/7 * (time spent in morning + afternoon + evening))  + (days in weekend/7 * (time spent in morning + afternoon + evening)) |
| Watching Television | 10 | “On average, how many days during the week does your child watch television/video/DVD?”   - - Never on weekdays (coded as 0)   - 1 day per week (coded as 1)   - 2 days per week (coded as 2)   - 3 days per week (coded as 3)   - 4 days per week (coded as 4)   - Every weekday (coded as 5)   “On average, how many weekend days in a week does your child watch television/video/DVD?”   - - Never in the weekend (coded as 0)   - 1 day in the weekend (coded as 1)   - 2 days in the weekend (coded as 2)   “On the days that your child watches television/video/DVD, how long does he watch, on average? Differentiate here between weekdays and weekends. Also, mornings/afternoons/evenings separately. “  *Separate questions for weekdays and weekends, and separately for mornings, afternoons, and evenings after dinner*   - - Never (coded as 0)   - Less than 30 minutes (coded as 0.25)   - 30-60 minutes (coded as 0.75)   - 1-2 hours (coded as 1.5)   - 2-3 hours (coded as 2.5)   - 3-4 hours (coded as 3.5) | Average time spent watching television per day =  (days during week/7 * (time spent in morning + afternoon + evening))  + (days in weekend/7 * (time spent in morning + afternoon + evening)) |
| Playing sports | 6 | “Does your child take part in sports (for example, football, judo, gymnastics, jazz ballet, tennis, etc.)? It is possible that your child plays more than 1 sport. You can fill in the following questions per sport.”   - “How many times per week does your child participate in this sport?”   - 1 time per week (coded as 1)   - 2 times per week (coded as 2)   - 3 times per week (coded as 3)   - More than 3 times per week (coded as 4) - “How long, on average, does your child participate in this sport each time (training session or match)?”   - Less than 30 minutes (coded as 0.5)   - 30 to 60 minutes (coded as 0.75)   - More than 1 hour (coded as 1.5) | Total time spent on three sports =  (amount of days sport 1 * time spent sport 1)  + (amount of days sport 2 * time spent sport 2)  + (amount of days sport 3 * time spent sport 3)  Total time categorized into three categories:  0: No sports participation  1: ≤ 1 hour/week sports participation  2: > 1 hour/week sports participation |
| Playing sports | 10 | “The following questions concern sports. By sports we mean organized sport activities taking place outside ‘normal’ school hours. Gym at school, therefore, does not count as sports.  How many hours per week does your child spend doing sports (training session and match/competition together)?”   - - Less than 1 hour per week (coded as 0.5)   - 1 to 2 hours per week (coded as 1.5)   - 2 to 4 hours per week (coded as 3)   - More than 4 hours per week (coded as 5) | No additional calculations |
| Activity limitation social | 6 | “During the past 4 weeks, was your child restricted in the type of schoolwork or activities with friends that he/she could undertake due to problems with his/her physical health?”   - - No, not restricted (coded as 0)   - Yes, somewhat restricted (coded as 1)   - Yes, restricted (coded as 2)   - Yes, very much restricted (coded as 3) | Activity limitation social categorized into two categories:  0: No, not restricted (= 0)  1: Yes, restricted (> 0) |
| Activity limitation physical | 6 | “During the past 4 weeks, was your child restricted in one of the following activities due to health problems?”   - “In activities that require a lot of effort, such as playing football or running?”   - No, not restricted (coded as 0)   - Yes, somewhat restricted (coded as 1)   - Yes, restricted (coded as 2)   - Yes, very much restricted (coded as 3) - “In activities that require some effort, such as cycling or (roller) skating?”   - No, not restricted (coded as 0)   - Yes, somewhat restricted (coded as 1)   - Yes, restricted (coded as 2)   - Yes, very much restricted (coded as 3) - “Bending, lifting or bending over?”   - No, not restricted (coded as 0)   - Yes, somewhat restricted (coded as 1)   - Yes, restricted (coded as 2)   - Yes, very much restricted (coded as 3) | Total sum of activity limitation physical categorized into two categories:  0: No, not restricted (= 0)  1: Yes, restricted (> 0) |
| Social interactions online | 10 | “How often does your child spend time chatting? By chatting we not only mean via MSN, but also via Websites such as Hyves, chat boxes, What’s App, Ping, etc.”   - - Never (coded as 0)   - 1 to 3 days per month (coded as 0.5)   - 1 to 3 days per week (coded as 2.5)   - 4 to 6 days per week (coded as 5)   - Every day (coded as 7)   “On the days your child chats on the computer, how long does he/she spend doing this per day?”   - - Less than 30 minutes per day (coded as 0.25)   - 30 minutes to 1 hour per day (coded as 0.75)   - 1 to 2 hours per day (coded as 1.5)   - More than 2 hours per day (coded as 2.5)   “How often does your child spend time on social network sites such as Hyves or Facebook?”   - - Never (coded as 0)   - 1 to 3 days per month (coded as 0.5)   - 1 to 3 days per week (coded as 2.5)   - 4 to 6 days per week (coded as 5)   - Every day (coded as 7)   “On the days that your child spends time on a social network sites, how long does he/she do this per day?”   - - Less than 30 minutes per day (coded as 0.25)   - 30 minutes to 1 hour per day (coded as 0.75)   - 1 to 2 hours per day (coded as 1.5)   - More than 2 hours per day (coded as 2.5) | Total time spent online for social purposes =  (days spent chatting * time spent chatting)  + (days spent on social media * time spent on social media)  Time spent online for social purposes categorized into two categories:  0: No (= 0)  1: Yes (> 0) |
| Social interactions outside of school | 10 | “How regular/regularly does your child get together with friends outside of school?”   - - Not at all (coded as 0)   - Slightly (coded as 1)   - Quite (coded as 2)   - Extremely (coded as 3) | No additional calculations |

**Supplementary Table 3** Associations of having a chronic physical condition and play behaviors with a binary measure of mental health problems at age 14 years, a sensitivity analysis

|  | **RR for CBCL borderline problems score at age 14 (95% CI)** | **p** |
| --- | --- | --- |
| **Chronic physical condition** |  |  |
| **No**  **Yes** | Reference  1.12 (0.90; 1.40) | 0.322 |
| **Playing outside (per hour/day)** |  |  |
| **Age 6** | 1.03 (0.95; 1.12) | 0.420 |
| **Age 10** | 0.98 (0.87; 1.12) | 0.809 |
| **Gaming (per hour/day)** |  |  |
| **Age 6** | 1.04 (0.87; 1.24) | 0.701 |
| **Age 10** | 1.02 (0.93; 1.13) | 0.652 |
| **Watching television (per hour/day)** |  |  |
| **Age 6** | 1.01 (0.92; 1.11) | 0.853 |
| **Age 10** | 1.03 (0.96; 1.10) | 0.474 |
| **Playing sports** |  |  |
| **Age 6**  **No ≤ 1 hour/week > 1 hour/week** | Reference  0.88 (0.72; 1.08) 0.94 (0.73; 1.20) | 0.231 0.606 |
| **Age 10**  **<1 hour/week 1-2 hours/week 2-4 hours/week >4 hours/week** | Reference  0.93 (0.62; 1.40) 0.73 (0.49; 1.09)  0.59 (0.39; 0.90) | 0.735 0.123 0.014* |
| **Activity limitation age 6** |  |  |
| **Social**  **No**  **Yes** | Reference  1.07 (0.77; 1.47) | 0.696 |
| **Physical**  **No**  **Yes** | Reference  1.07 (0.80; 1.44) | 0.631 |
| **Social interactions age 10** |  |  |
| **Online**  **No**  **Yes** | Reference  1.09 (0.91; 1.32) | 0.340 |
| **Outside of school**  **Not at all**  **Slightly Quite Extremely** | Reference  0.74 (0.52; 1.06) 0.47 (0.33; 0.66) 0.46 (0.32; 0.66) | 0.098 <0.001* <0.001* |

Abbreviation: RR, risk ratio; CBCL, Child Behavior Checklist

A binary score indicating borderline problematic issues on the CBCL was calculated (for boys aged 12-18 years, a score of ≥40 was considered borderline problematic, and for girls aged 12-18 years, a score of ≥36 was considered borderline problematic). All models were quasi-Poisson regression analyses adjusted for age at the measuring point of the CBCL, sex, ethnicity, maternal educational level, and maternal psychopathology. Moreover, models involving play behaviors as predictors were also adjusted for CBCL score measured at age 6

**Supplementary Table 4** Associations of having a chronic physical condition and play behaviors with mental health problems at age 10 years, a sensitivity analysis

|  | **Estimate for CBCL total problems score at age 10 (95% CI)** | **p_uncorrected_** | **p_adjusted_** | **Effect size partial R^2^** |
| --- | --- | --- | --- | --- |
| **Chronic physical condition** |  |  |  |  |
| **No**  **Yes** | Reference  0.30 (0.16; 0.44) | <0.001* | <0.001* | 0.0045 |
| **Playing outside (per hour/day)** |  |  |  |  |
| **Age 6** | -0.03 (-0.07; 0.02) | 0.260 | 0.510 | 0.00029 |
| **Age 10** | -0.06 (-0.13; 0.01) | 0.107 | 0.284 | 0.00058 |
| **Gaming (per hour/day)** |  |  |  |  |
| **Age 6** | -0.06 (-0.18; 0.06) | 0.315 | 0.510 | 0.00034 |
| **Age 10** | 0.04 (-0.03; 0.11) | 0.227 | 0.485 | 0.00037 |
| **Watching television (per hour/day)** |  |  |  |  |
| **Age 6** | -0.03 (-0.09; 0.03) | 0.278 | 0.510 | 0.00031 |
| **Age 10** | 0.07 (0.02; 0.12) | 0.004* | 0.014* | 0.00181 |
| **Playing sports** |  |  |  |  |
| **Age 6**  **No ≤ 1 hour/week > 1 hour/week** | Reference  -0.06 (-0.17; 0.06) 0.02 (-0.10; 0.15) | 0.344 0.736 | 0.516 0.903 | 0.00033 |
| **Age 10**  **<1 hour/week 1-2 hours/week 2-4 hours/week >4 hours/week** | Reference  0.00 (-0.24; 0.25) -0.15 (-0.40; 0.09)  -0.28 (-0.53; -0.03) | 0.983 0.218 0.031* | 0.983 0.485 0.099 | 0.00366 |
| **Activity limitation age 6** |  |  |  |  |
| **Social**  **No**  **Yes** | Reference  0.21 (0.01; 0.41) | 0.043* | 0.126 | 0.00084 |
| **Physical**  **No**  **Yes** | Reference  0.11 (-0.07; 0.30) | 0.214 | 0.485 | 0.00037 |
| **Social interactions age 10** |  |  |  |  |
| **Online**  **No**  **Yes** | Reference  0.01 (-0.09; 0.11) | 0.867 | 0.957 | 0.00002 |
| **Outside of school**  **Not at all**  **Slightly Quite Extremely** | Reference  -0.42 (-0.70; -0.13) -0.69 (-0.96; -0.43) -0.87 (-1.14; -0.60) | 0.004* <0.001* <0.001* | 0.016* <0.001* <0.001* | 0.01347 |

Abbreviation: CBCL, Child Behavior Checklist

CBCL outcome was square root transformed. All models were linear regression analyses adjusted for age at the measuring point of the CBCL, sex, ethnicity, maternal educational level, and maternal psychopathology. Moreover, models involving play behaviors as predictors were also adjusted for CBCL score measured at age 6

The effect size partial R^2^ represents the explained variance of the CBCL score by the specified predictor (having a chronic physical condition or a play behavior) in the presence of the covariates. The effect size measure partial R^2^ can be interpreted as small (0.02), medium (0.13), or large (0.26) [2]

**References**

[1] Visser AM, Jaddoe VWV, Arends LR, Tiemeier H, Hofman A, Moll HA, et al. Paroxysmal disorders in infancy and their risk factors in a population-based cohort: The Generation R Study. Dev Med Child Neurol 2010;52:1014–20. https://doi.org/10.1111/j.1469-8749.2010.03689.x.

[2] Cohen J. Statistical power analysis for the behavioral sciences. 2nd ed. Hillside, NJ: Lawrence Erlbaum Associates; 1988.
